# FIN219/JAR1 and cryptochrome1 antagonize each other to modulate photomorphogenesis under blue light in *Arabidopsis*

**DOI:** 10.1371/journal.pgen.1007248

**Published:** 2018-03-21

**Authors:** Huai-Ju Chen, Tsu-Yu Fu, Shao-Li Yang, Hsu-Liang Hsieh

**Affiliations:** Institute of Plant Biology, College of Life Science, National Taiwan University, Taipei, Taiwan; Peking University, CHINA

## Abstract

Plant development is affected by the integration of light and phytohormones, including jasmonates (JAs). To address the molecular mechanisms of possible interactions between blue light and JA signaling in *Arabidopsis thaliana*, we used molecular and transgenic approaches to understand the regulatory relationships between FAR-RED INSENSITIVE 219 (FIN219)/JASMONATE RESISTANT1 (JAR1) and the blue-light photoreceptor cryptochrome1 (CRY1). *FIN219* overexpression in the wild type resulted in a short-hypocotyl phenotype under blue light. However, *FIN219* overexpression in *cry1*, *cry2* and *cry1cry2* double mutant backgrounds resulted in phenotypes similar to their respective mutant backgrounds, which suggests that FIN219 function may require blue light photoreceptors. Intriguingly, *FIN219* overexpression in transgenic plants harboring ectopic expression of the C terminus of CRY1 (GUS-CCT1), which exhibits a hypersensitive short-hypocotyl phenotype in all light conditions including darkness, led to a rescued phenotype under all light conditions except red light. Further expression studies showed mutual suppression between FIN219 and CRY1 under blue light. Strikingly, *FIN219* overexpression in *GUS-CCT1* transgenic lines (*FIN219-OE/GUS-CCT1*) abolished GUS-CCT1 fusion protein under blue light, whereas GUS-CCT1 fusion protein was stable in the *fin219-2* mutant background (*fin219-2/GUS-CCT1*). Moreover, FIN219 strongly interacted with COP1 under blue light, and methyl JA (MeJA) treatment enhanced the interaction between FIN219 and GUS-CCT1 under blue light. Furthermore, FIN219 level affected *GUS-CCT1* seedling responses such as anthocyanin accumulation and bacterial resistance under various light conditions and MeJA treatment. Thus, FIN219/JAR1 and CRY1 antagonize each other to modulate photomorphogenic development of seedlings and stress responses in Arabidopsis.

## Author summary

The crosstalks between light and plant hormones are critical in plant growth and development as well as stress responses. This study reveals the interaction between blue light and jasmonate (JA) signaling via direct interaction of the photoreceptor CRY1 and a JA-conjugating enzyme FIN219/JAR1. FIN219 function in blue light requires the functional blue light photoreceptor CRY1. Gene expression and genetic studies showed a mutually antagonistic relationship between FIN219 and CRY1, which suggests that a critical balance between blue light and JA signaling is important for seedling development and stress responses. Intriguingly, FIN219-mediated rescue of the *GUS-CCT1* seedlings was due to degradation of GUS-CCT1 fusion protein as a result of FIN219 interaction with GUS-CCT1 under blue light, which likely involves increased activity of COP1 and reduced levels of HY5. Thus, our studies reveal an antagonistic link between blue light and JA signaling pathways in regulating seedling development and stress responses in Arabidopsis.

## Introduction

Integration of light and phytohormones affects many aspects of plant growth and development, including seed germination [1, 2], hypocotyl elongation [3–6] and defense responses [7–9]. The molecular mechanisms underlying the interaction leading to physiological responses have been revealed recently [10–12]. Light-activated phytochromes enhance seed germination by negatively regulating PHYTOCHROME-INTERACTING FACTOR3-LIKE5 (PIL5)-mediated activation of *GA2ox2*, DELLA and abscisic acid biosynthetic genes [13]. Light integrates with almost all known phytohormones to modulate hypocotyl elongation of seedling development [7]. Recent evidence has revealed a vital role for a light-mediated dynamic balance between plant development and defense responses in regulating the early development of seedlings [8, 14, 15]. However, the molecular mechanisms underlying the interaction of monochromatic light such as blue light and jasmonates (JAs) remain poorly understood.

FAR-RED INSENSITIVE 219/JASMONATE RESISTANT1 (FIN219/JAR1) participates in far-red (FR) light signaling [3, 16, 17] and functions as a JA-conjugating enzyme responsible for the formation of a physiologically active form, JA-isolecucine (JA-Ile) [17]. Ectopic expression of *FIN219* in wild-type Columbia (Col-0) resulted in a shorter hypocotyl phenotype than in Col-0 under blue light [3, S1 Fig], which suggests that FIN219 may play a role in blue light. In addition, under FR light, FIN219 interacts with CONSTITUTIVE PHOTOMORPHOGENIC 1 (COP1), a repressor of photomorphogenesis in the dark [18]. Further studies indicated that FIN219 regulates the levels of COP1 negatively and HY5 positively [18].

The blue-light photoreceptors, cryptochromes cry1 and cry2, regulate hypocotyl elongation and flowering in response to blue-light irradiation [19, 20]. Ectopic expression of the C-terminal domain of CRY1 or CRY2 in Col-0 (GUS-CCT1 or GUS-CCT2) resulted in a short hypocotyl phenotype under all light conditions, including the dark, which is similar to the *cop1* mutant phenotype [21]. Further studies revealed that the *cop1*-like phenotype caused by *GUS-CCT1* overexpression was due to the interaction of GUS-CCT1 and COP1, which led to a release of HY5 and photomorphogenic development [22, 23]. Thus, whether FIN219/JAR1 plays a role in blue-light signaling and has a regulatory relationship with CRY1 remains to be elucidated.

Another signaling component, SUPPRESSOR OF PHYA-105 (SPA1), is a repressor of phytochrome A-mediated responses in FR light [24]. SPA1 interacts with COP1 to downregulate HY5 levels, which leads to reduced photomorphogenesis [25]. SPA1 can interact with CRY1 to suppress COP1 activity in response to blue light [26]. Moreover, FIN219 negatively regulates *SPA1* transcript levels [18]. Whether FIN219 affects the relative relations among CRY1, COP1 and SPA1 in response to blue light remains elusive.

Here we investigated the regulatory relationship between FIN219 and CRY1 by introducing *FIN219* overexpression in *GUS-CCT1* transgenic plants with blue light and JA treatment. FIN219 and CRY1 negatively regulated each other by direct interaction in response to JA under blue light. We reveal a vital mechanism in the integration of blue light and JA signaling to control seedling development in *Arabidopsis*.

## Results

### Ectopic expression of *FIN219* in cryptochrome mutants reveals a functional requirement for blue-light photoreceptors for FIN219 function

**Fig 1:**
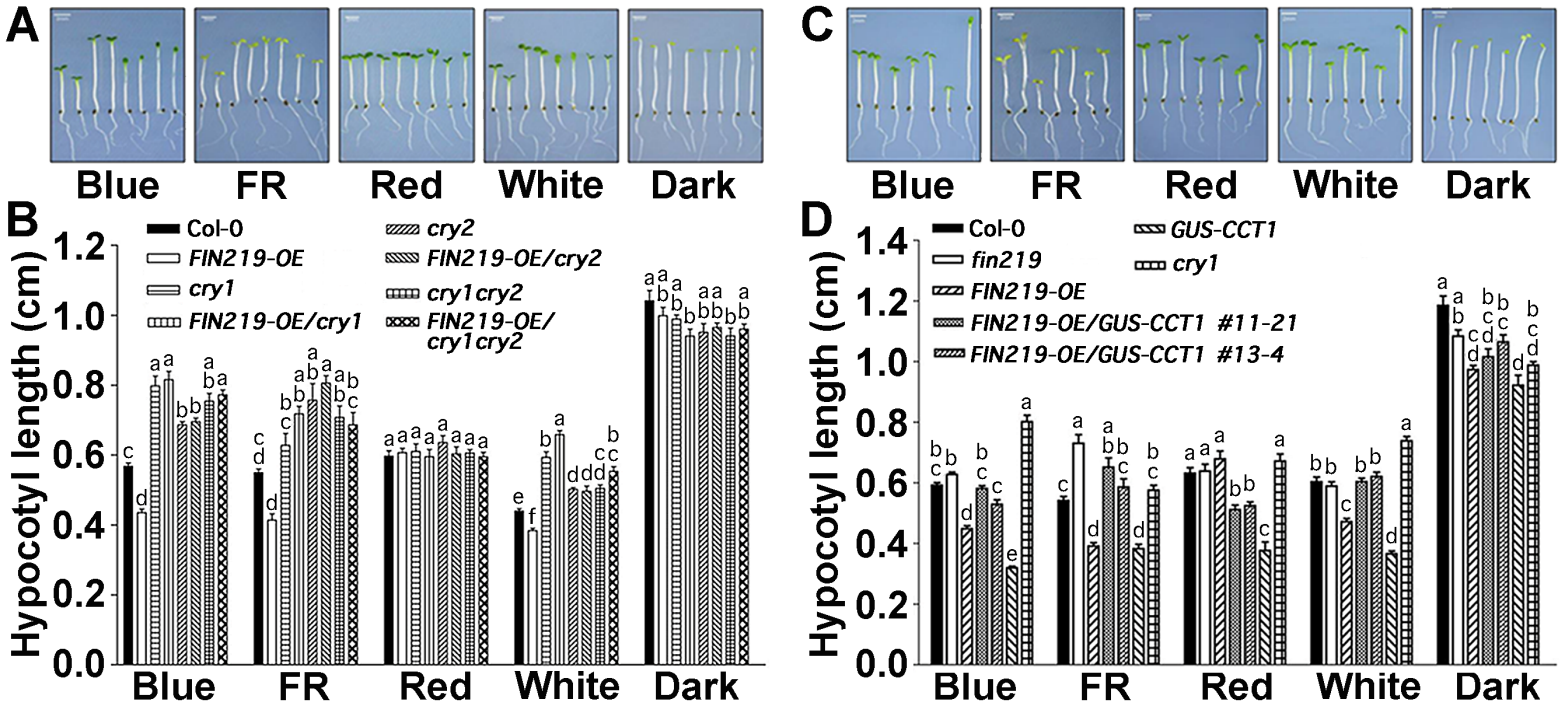
FIN219 and CRY1 regulate each other in modulating hypocotyl elongation of *Arabidopsis* seedlings under different light conditions. (A) FIN219 function in regulating light-mediated inhibition of hypocotyl elongation requires functional CRY1 and CRY2. Hypocotyl growth responses of 4-day-old seedlings under different light conditions. (Left to right) Wild type (Col-0), *FIN219-OE*, *cry1*, *FIN219-OE/cry1*, *cry2*, *FIN219-OE/cry2*, *cry1cry2* and *FIN219-OE/cry1cry2*. Blue light: 2.2 μmol•m^-2^•s^-1^, far-red light (FR): 3.1 μmol•m^-2^•s^-1^, red light: 7.5 μmol•m^-2^•s^-1^, white light: 8 μmol•m^-2^•s^-1^. Scale bar = 2 mm. (B) Quantification of hypocotyl lengths of seedlings shown in (A). Data are mean ± SD (n ≥ 20). Different lowercase letters represent significant differences by one-way ANOVA at P <0.05. (C) *FIN219* overexpression in *GUS-CCT1* transgenic plants can rescue the short-hypocotyl phenotype of *GUS-CCT1* under all light conditions except red light. Hypocotyl growth responses of 4-day-old seedlings under various light conditions, including the dark. (Left to right) wild type (Col-0), *fin219*, *FIN219-OE*, *FIN219-OE/GUS-CCT1 #11–21*, *#13–4*, *GUS-CCT1* and *cry1*. Blue light: 2.2 μmol•m^-2^•s^-1^, far-red light (FR): 3.1 μmol•m^-2^•s^-1^, red light: 7.5 μmol•m^-2^•s^-1^, white light: 8 μmol•m^-2^•s^-1^. Scale bar = 2 mm. (D) Quantification of hypocotyl lengths of seedlings shown in C. Data are mean ± SD (n ≥ 20). Different lowercase letters represent significant differences by one-way ANOVA at P <0.05.

We crossed *FIN219*-overexpressing lines (*FIN219-OE*) with *cry1*, *cry2* and *cry1cry2* mutants and examined the phenotypes of the resulting homozygous transgenic lines (*FIN219-OE*/*cry1*, *FIN219-OE*/*cry2*, and *FIN219-OE*/*cry1cry2*) under different light conditions. *FIN219-OE*/*cry1*, *FIN219-OE/cry2*, and *FIN219-OE/cry1cry2* seedlings exhibited a long-hypocotyl phenotype similar to the respective genetic backgrounds under blue, FR and white light conditions (Fig 1A and 1B), which suggests that the FIN219 function under blue light may require functional CRY1 and CRY2. FIN219 is mainly responsible for the formation of a physiologically active form of JA-Ile [27], but how FIN219 functions with CRY1 and CRY2 remains elusive. CRY1 has been implicated in promoting R protein-mediated plant resistance to *Pseudomonas syringae* in Arabidopsis [28]. CRY1 was also shown to function in drought response [29], and CRY1 and CRY2 are involved in osmotic stress response in wheat [30]. Thus, stress responses mediated by blue-light photoreceptor cryptochrome may involve JA-mediated signaling.

### *FIN219* overexpression in *GUS-CCT1* transgenic plants can rescue the short-hypocotyl phenotype of *GUS-CCT1* under all light conditions except red light

To further elucidate the FIN219 functional relationship with CRY1 in blue-light signaling, we crossed *FIN219-OE* lines with *GUS-CCT1* transgenic lines ectopically expressing the C terminus of CRY1 in a Col-0 background and obtained *FIN219-OE/GUS-CCT1* transgenic seedlings. *FIN219-OE/GUS-CCT1* transgenic seedlings showed a rescued phenotype similar to Col-0 under blue, FR, white light, and dark conditions and only a partially rescued phenotype, with intermediate hypocotyl length, as compared to Col-0 and *GUS-CCT1* under red light (Fig 1C and 1D), so FIN219 may need other factors to complement the *GUS-CCT1* phenotype in red light.

### FIN219 and CRY1 antagonize each other under blue light

**Fig 2:**
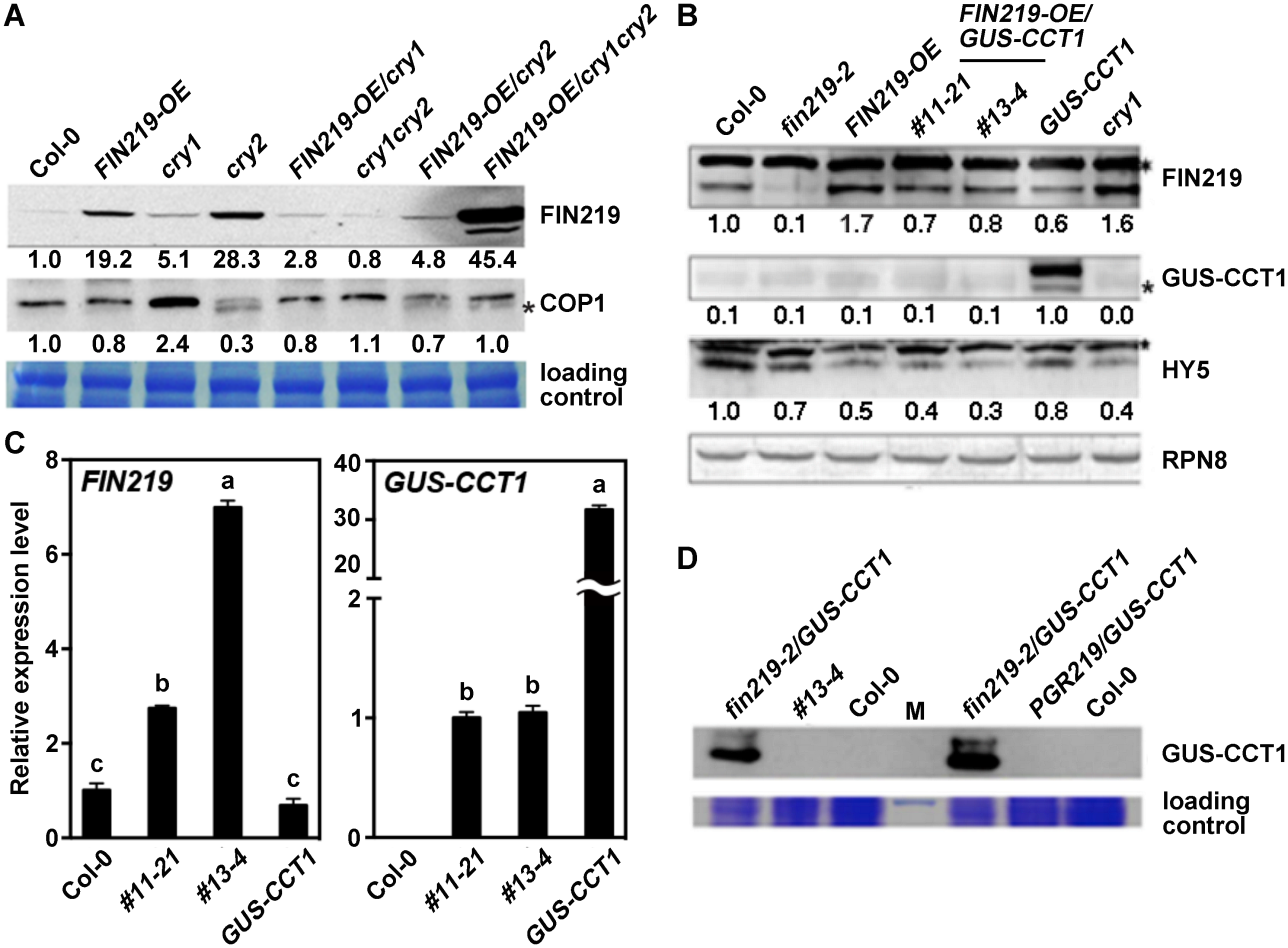
FIN219 and CRY1 antagonize each other under blue light. (A) CRY1 and CRY2 negatively regulate FIN219 protein level under blue light. Western blot analysis of FIN219 protein level in wild-type Col-0, *cry1*, *cry2* mutants and transgenic seedlings grown under blue light. The signal was detected by FIN219 monoclonal antibody. Blue light: 2.2 μmol•m^-2^•s^-1^. The number below each blot represents the level of the indicated protein. The level of wild-type Col-0 was arbitrarily set to 1. The asterisk (*) indicates nonspecific bands. (B) *FIN219* overexpression in *GUS-CCT1* seedlings abolishes GUS-CCT1 fusion protein in blue light. Western blot analysis of protein levels in Col-0, *fin219-2*, *FIN219* overexpression line (*FIN219-OE*), *FIN219-OE/GUS-CCT1*, *GUS-CCT1* and *cry1* seedlings grown in blue light for 3 days. The blots were detected by antibodies against FIN219 and GUS-CCT1 and HY5. Blue light: 2.2 μmol•m^-2^•s^-1^. RPN8 was a loading control. The asterisk (*) indicates nonspecific bands. The number below each blot represents the level of the indicated protein. The level of wild-type Col-0 was arbitrarily set to 1. (C) *GUS-CCT1* transcripts detected in transgenic seedlings of *FIN219-OE/GUS-CCT1* under blue light. Quantitative Real-time PCR (qPCR) analysis of transgenic seedlings shown in B. Total RNAs were extracted from transgenic seedlings shown in the figure and subjected for qPCR analysis. *Ubiquitin 10* (*UBQ10*) was an internal control. (D) GUS-CCT1 fusion proteins were stable in *fin219-2/GUS-CCT1* seedlings under blue light. Western blot analysis of GUS-CCT1 level in Col-0, *FIN219-OE/GUS-CCT1* (#13–4), *fin219-2/GUS-CCT1* and *PGR219/GUS-CCT1* seedlings grown in blue light for 4 days. Total proteins extracted from seedlings were probed with GUS antibody. M, protein size markers.

To understand the molecular mechanisms underlying the phenotypic responses of *FIN219-OE/cry1*, *FIN219-OE/cry2*, and *FIN219-OE/cry1cry2* seedlings under blue light, we examined FIN219 protein level in these seedlings under blue and FR light. Under blue light, FIN219 level increased in the *cry1* mutant and only slightly increased in the *cry2* mutant (Fig 2A and 2B). In contrast, FIN219 level was substantially greater in *FIN219-OE*/*cry1* than *FIN219-OE* seedlings, which suggests that CRY1 negatively regulates FIN219 protein level. However, FIN219 level was significantly lower in *FIN219-OE*/*cry2* than *FIN219-OE* seedlings (Fig 2A), so the CRY2 effect on FIN219 levels might involve mechanisms different from those of CRY1.

FIN219 protein level was strikingly increased in *FIN219-OE*/*cry1cry2* seedlings (Fig 2A). Hence, under blue light, *FIN219* overexpression in the *cry1cry2* double mutant may suppress other negative regulators, both CRY1 and CRY2 may negatively regulate FIN219 levels, or the substantial increase in FIN219 levels in *cry1cry2* may involve unknown mechanisms, not leading to photomorphogenic development for *FIN219-OE*/*cry1cry2* under blue light (Fig 1A). In addition, under FR light, CRY1 negatively regulated FIN219, but CRY2 played a minor role in modulating FIN219 level (S2 Fig). Moreover, FIN219 level in the *cry2* mutant and other mutant backgrounds remained largely the same as in Col-0 (S2 Fig). Thus, CRY1 negatively regulated FIN219 levels under both blue and FR light conditions.

Since *FIN219* overexpression in *GUS-CCT1* seedlings resulted in a rescued phenotype under most of the light conditions examined, we further examined FIN219 protein level in *FIN219-OE/GUS-CCT1* seedlings. Under blue light, FIN219 level was lower in *GUS-CCT1* than wild-type Col-0 seedlings and was higher in *FIN219-OE/GUS-CCT1* than *GUS-CCT1* seedlings (Fig 2B) but was lower in *FIN219-OE/GUS-CCT1* than *FIN219-OE* seedlings (Fig 2B). The finding is consistent with CRY1 negatively regulating FIN219 under blue light (Fig 2A). Surprisingly, GUS-CCT1 fusion proteins were not detected in *FIN219-OE/GUS-CCT1* seedlings but were greatly expressed in *GUS-CCT1* seedlings (Fig 2B). Quantitative real-time PCR (qPCR) and RT-PCR analyses detected *GUS-CCT1* transcripts in *FIN219-OE/GUS-CCT1* seedlings (Fig 2C; S3 Fig), which suggests that the disappearance of GUS-CCT1 fusion protein may involve posttranscriptional regulation under blue light. Moreover, the transcript levels of *GUS-CCT1* in two independent transgenic lines #11–21 and #13–4 of *FIN219-OE/GUS-CCT1* were much lower than that in *GUS-CCT1* line. Conversely, *FIN219* transcript levels in both #11–21 and #13–4 were greatly abundant compared to Col-0 and *GUS-CCT1* line (Fig 2C; S3 Fig). Taken together, the mutual regulation between FIN219 and CRY1 may involve transcriptional as well as posttranscriptional levels.

Thus, we further crossed the *GUS-CCT1* transgenic line with the *fin219-2* mutant and obtained *fin219-2/GUS-CCT1* lines to determine whether GUS-CCT1 fusion proteins exist in a *fin219* mutant background. In addition, we generated a new line, *PGR219/GUS-CCT1*, by crossing the *GUS-CCT1* transgenic line with the inducible *FIN219*-overexpressing line *PGR219* to confirm the GUS-CCT1 fusion proteins. GUS-CCT1 fusion protein levels were stably accumulated in *fin219-2/GUS-CCT1* seedlings but not *FIN219-OE/GUS-CCT1* or *PGR219/GUS-CCT1* seedlings (Fig 2D). Thus, *FIN219* overexpression in *GUS-CCT1* seedlings resulted in undetected levels of the GUS-CCT1 fusion proteins under blue light.

COP1 interacts with GUS-CCT1 in the dark and under blue light [22, 23]. As well, COP1 is negatively regulated by FIN219 [18]. Here, we found that FIN219 negatively regulated COP1 under blue light (Fig 2A; S4 Fig). However, COP1 level in *GUS-CCT1* and *FIN219-OE/GUS-CCT1* was greater than that in *FIN219-OE* line, which suggests that COP1 may be modulated by GUS-CCT1 as shown with increased COP1 levels in the *cry1* mutant (S4 Fig). Level of HY5, a positive regulator in photomorphogenesis, was significantly reduced in all samples examined under blue light as compared with Col-0 (Fig 2B). Previous study indicated that FIN219 positively modulated HY5 levels under FR light. Of note, HY5 was downregulated in the *fin219-2* mutant as compared to Col-0 and was greatly reduced in *FIN219*-*OE* lines under blue light (Fig 2B). Therefore, FIN219-regulated HY5 levels under blue light may involve other factors to modulate seedling development likely through COP1.

### Degradation of GUS-CCT1 fusion proteins in *FIN219-OE/GUS-CCT1* seedlings was mediated by 26S proteasome under blue light

**Fig 3:**
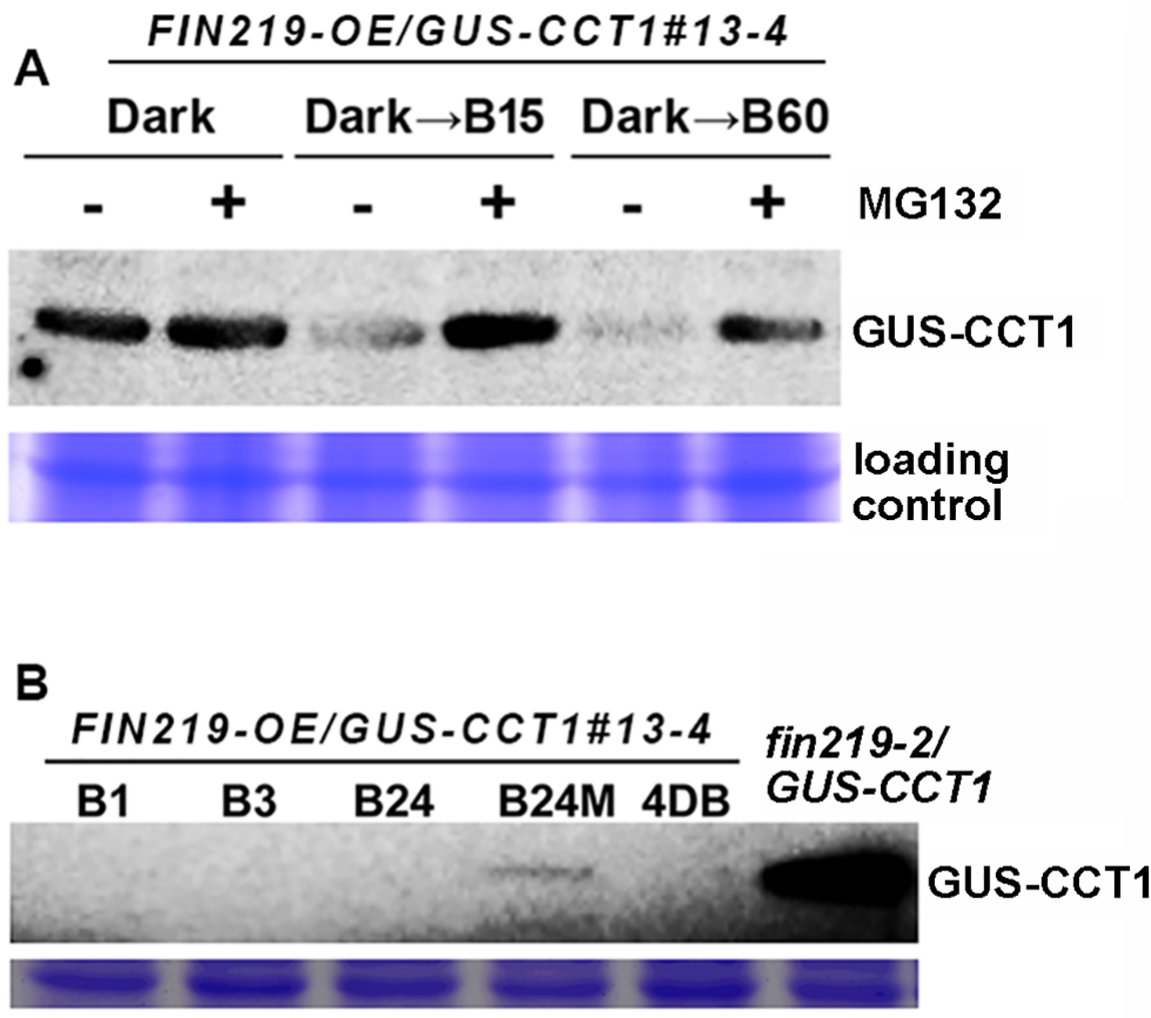
Degradation of GUS-CCT1 fusion proteins in *FIN219-OE/GUS-CCT1* seedlings is mediated by 26S proteasome under blue light. (A) Western blot analysis of GUS-CCT1 level in *FIN219-OE/GUS-CCT1* seedlings transferred from the dark to blue light for different times (B15: 15 min blue light; B60: 60 min blue light) as shown above the figure. “+”: with MG132; “—”: without MG132. (B) Western blot analysis of GUS-CCT1 protein level in *FIN219-OE/GUS-CCT1* seedlings transferred from the dark to blue light for different times shown above the figure. *fin219-2/GUS-CCT1* seedlings were grown for 4 days under blue light. B1: 1-h blue light; B3: 3-h blue light; B24: 24-h blue light; B24M: 24-h blue light plus MG132; 4DB: 4 days of blue light.

We further examined whether degradation of GUS-CCT1 fusion proteins was mediated by the ubiquitin/26S proteasome system. We performed light transition studies by transferring *FIN219-OE/GUS-CCT1* seedlings from darkness to blue light for various times with or without the 26S proteasome inhibitor MG132. GUS-CCT1 fusion proteins were stably present in the dark and greatly reduced at 60 min under blue light (Fig 3A) and barely detected at 1 h or longer under blue light (Fig 3B). In contrast, MG132 addition could efficiently stabilize GUS-CCT1 fusion protein level under 15- and 60-min blue-light exposure (Fig 3A), so the degradation of GUS-CCT1 fusion proteins was mediated by 26S proteasome and occurred rapidly under blue light.

### Exogenous methyl JA (MeJA) can enhance FIN219 levels in GUS-CCT1 seedlings especially in blue light

**Fig 4:**
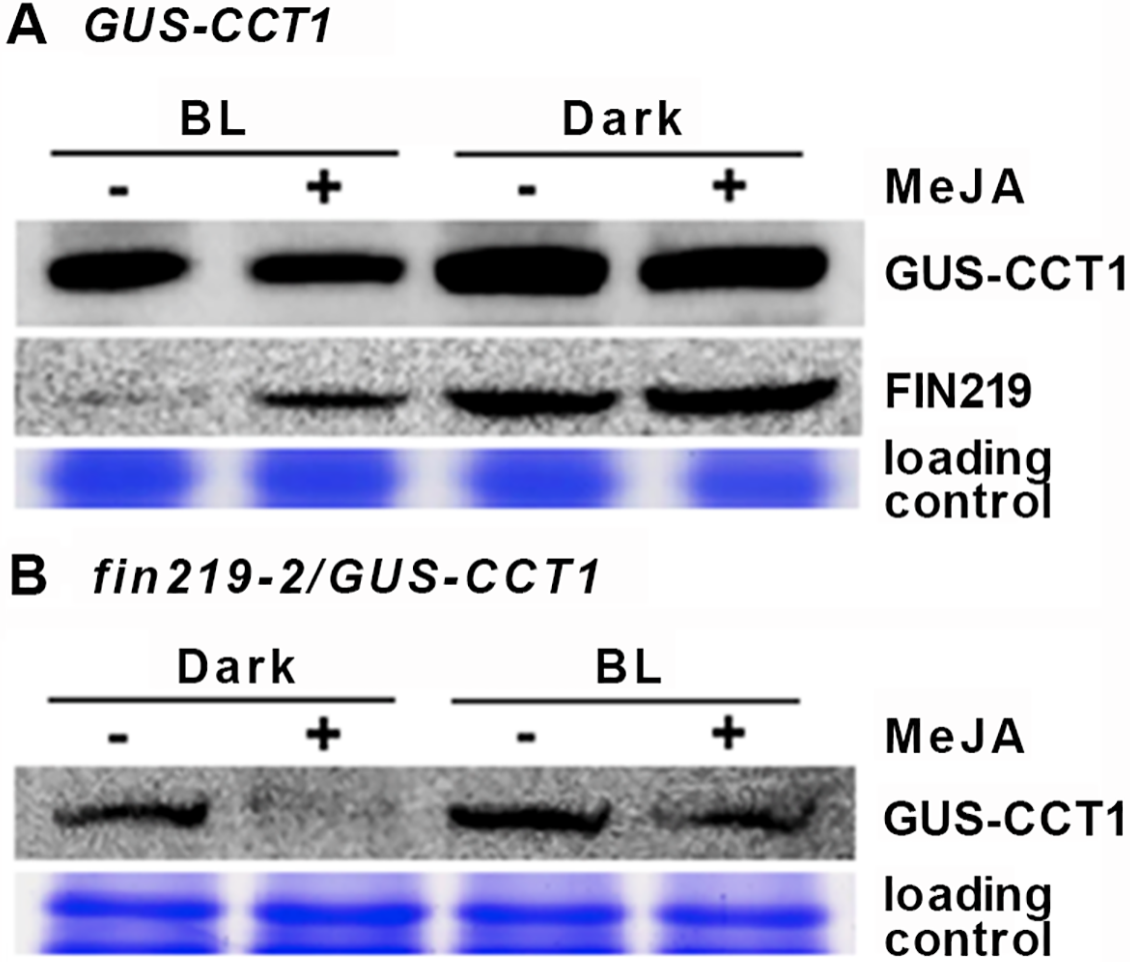
FIN219 affects GUS-CCT1 stability under the dark and blue light. (A) Exogenous MeJA slightly reduced GUS-CCT1 level in *GUS-CCT1* seedlings under continuous blue light and dark. Western blot analysis of GUS-CCT1 and FIN219 protein levels in *GUS-CCT1* seedlings grown in continuous blue light and the dark without or with MeJA for 3 days. (B) Exogenous MeJA greatly decreased GUS-CCT1 level in *fin219-2/GUS-CCT1* seedlings under continuous blue light and dark. Western blot analysis of GUS-CCT1 level in *fin219-2/GUS-CCT1* seedlings grown in dark and continuous blue light without or with MeJA for 3 days. GUS-CCT1 was detected by antibodies against GUS.

*FIN219* overexpression in *GUS-CCT1* seedlings (*FIN219-OE/GUS-CCT1*) led to degradation of GUS-CCT1 fusion protein under blue light (Figs 2B, 2D and 3A). This observation raises two possibilities: the degradation of GUS-CCT1 protein is caused by 1) JA-Ile, because overexpression of FIN219 produces more JA-Ile in cells, or 2) protein–protein interactions triggered by *FIN219* overexpression. To test the first possibility, we examined the effect of exogenous MeJA on GUS-CCT1 fusion proteins in *GUS-CCT1* seedlings under blue light and dark. GUS-CCT1 protein level was stable under both light conditions without MeJA treatment. MeJA slightly reduced GUS-CCT1 level under both light conditions (Fig 4A). In contrast, FIN219 level in *GUS-CCT1* seedlings was greatly reduced under blue light as compared to the dark; however, MeJA could greatly enhance FIN219 level under blue light but only slightly in the dark (Fig 4A).

We further examined the level of GUS-CCT1 in *fin219-2/GUS-CCT1* seedlings. GUS-CCT1 level was present in the dark and increased under blue light as compared to the dark; MeJA addition substantially reduced GUS-CCT1 level in *fin219-2/GUS-CCT1* seedlings in the dark, with a marked decrease under blue light as compared to without MeJA (Fig 4B). Thus, MeJA with the conversion to JA-Ile in cells may not be the major factor in the degradation of GUS-CCT1 protein in *FIN219-OE/GUS-CCT1* seedlings under blue light. Protein–protein interaction mediated by FIN219 overexpression is likely mainly responsible for the degradation.

### MeJA treatment enhances FIN219 and CRY1 interaction under blue light

**Fig 5:**
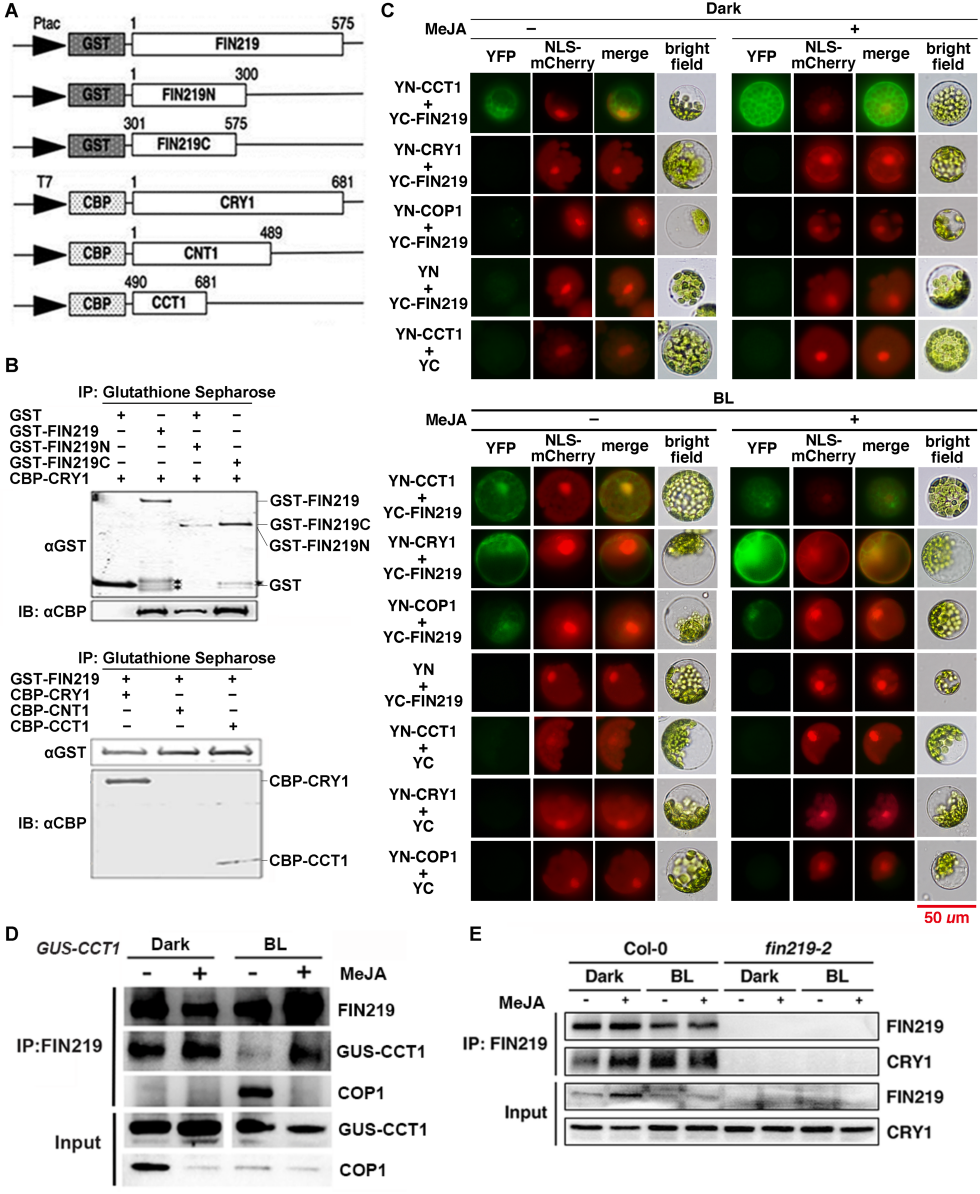
MeJA greatly enhanced FIN219 and GUS-CCT1 interaction under continuous blue light. (A) Schematic diagrams of different constructs corresponding to the full-length FIN219 (GST-FIN219), the N terminus and C terminus of FIN219 (GST-FIN219N and GST-FIN219C) and the full-length cryptochrome CRY1 (CBP-CRY1), the N terminus and C terminus of CRY1 (CBP-CNT1 and CBP-CCT1). (B) Pull-down assay of FIN219 interacting with CRY1 via its C terminus. Recombinant proteins GST, GST-FIN219, GST-FIN219N or GST-FIN219C were mixed with CBP-CRY1 and underwent protein pull-down assays (left panel). The asterisk (*) indicates nonspecific bands. The arrowhead represents GST-FIN219C. Right panel: Recombinant proteins CBP-CRY1, CBP-CNT, or CBP-CCT1 were mixed with GST-FIN219 and underwent protein pull-down assays. The mixtures were immunoprecipitated with glutathione sepharose for GST-tag, then probed with antibodies against CBP-tag. Upper and lower arrowheads represent CBP-CRY1 and CBP-CCT1, respectively. (C) BiFC assays showing FIN219 and CRY1 or COP1 interaction in the dark (top panel) and blue light (BL) (bottom panel). The protoplasts isolated from short-day grown Col-0 were transfected with YN-CRY1 or COP1 and YC-FIN219 without (–) or with (+) 50 μM MeJA treatment. Blue light (BL): 2.2 μmol•m^-2^•s^-1^. (D) Co-immunoprecipitation assay showing FIN219 and GUS-CCT1 interaction greatly enhanced by MeJA under blue light. GUS-CCT1 transgenic seedlings were grown in the dark and blue light for 4 days with (+) or without (–) MeJA. Total proteins 2 mg extracted from seedlings were immunoprecipitated with FIN219 monoclonal antibodies, then probed with GUS and COP1 polyclonal antibodies. (E) Co-immunoprecipitation assay showing FIN219 and CRY1 interaction greatly enhanced by MeJA under the dark. Wild-type Col-0 and *fin219-2* seedlings were grown in the dark and blue light for 4 days with (+) or without (–) MeJA. Total proteins 2 mg extracted from seedlings were immunoprecipitated with FIN219 monoclonal antibodies, and then probed with CRY1 polyclonal antibodies.

To further test the possibility of FIN219 and CRY1 interaction, we performed *in vitro* pull-down assays with the recombinant proteins FIN219 full-length (GST-FIN219) and the N and C terminus of FIN219 (GST-FIN219N and GST-FIN219C, respectively) as well as the recombinant proteins CRY1 full-length (CBP-CRY1) and N terminus (CNT1) and C terminus (CCT1) of CRY1 (Fig 5A). FIN219 could interact with CBP-CRY1 and CCT1 with higher affinity via its C than N terminus (Fig 5B). CRY1 proteins from different species show a light-dependent nucleocytoplasmic shuttling pattern [21, 30, 31] and FIN219 is mainly a cytoplasmic protein [3]. To further confirm the interaction of FIN219 and CRY1, bimolecular fluorescence complementation (BiFC) assays under the dark revealed that FIN219 interacted with CCT1, rather than CRY1 in both the cytoplasm and the nucleus and MeJA addition can enhance their interaction in the whole cell (Fig 5C, top panel). In contrast, FIN219 could interact with both CCT1 and CRY1 under blue light (Fig 5C, bottom panel), which suggests that FIN219 interacts with the photoactivated CRY1. FIN219 also interacted with COP1 under blue light, but did not under the dark, without or with MeJA (Fig 5C). Further co-immunoprecipitation (Co-IP) studies were performed with *GUS-CCT1* seedlings grown in the dark and blue light with or without MeJA treatment. Indeed, FIN219 could interact with GUS-CCT1 in the dark, but this interaction was greatly reduced under blue light, likely because of a strong interaction between FIN219 and COP1 under the same condition (Fig 5D). Intriguingly, MeJA could greatly enhance the FIN219 and GUS-CCT1 interaction, especially under blue light, but largely abolished the FIN219 and COP1 interaction (Fig 5D). To validate the full-length CRY1 and FIN219 interaction in wild-type Col-0, Co-IP assays further indicated that FIN219 did interact with CRY1 in Col-0 with less intensity under the dark than blue light. MeJA addition could greatly increase their interaction under the dark and also lead to their interaction with similar intensity to the blue light alone (Fig 5E). The discrepancy between FIN219 and CRY1 interacting affinity detected by BiFC and Co-IP is likely due to protein levels as well as tight regulation in response to blue light and MeJA (Fig 5C–5E). Alternatively, the exposed C terminus of CRY1 (CCT1) may have higher affinity with its interacting partners such as COP1 than the full-length CRY1. MeJA-induced FIN219 likely competitively binds with GUS-CCT1 under blue light (Fig 5D). It seems that the photoactivated full-length CRY1 can interact with FIN219 via its C terminus (GUS-CCT1) under blue light (Fig 5D and 5E). Thus, *FIN219* overexpression in *GUS-CCT1* seedlings, likely leading to more JA-Ile, may enhance the FIN219 and GUS-CCT1 interaction under blue light, thereby increasing COP1 association with the FIN219 and GUS-CCT1 complex to cause the degradation of GUS-CCT1 protein.

### FIN219 level regulates CRY1 activities in response to blue light and bacterial pathogens

**Fig 6:**
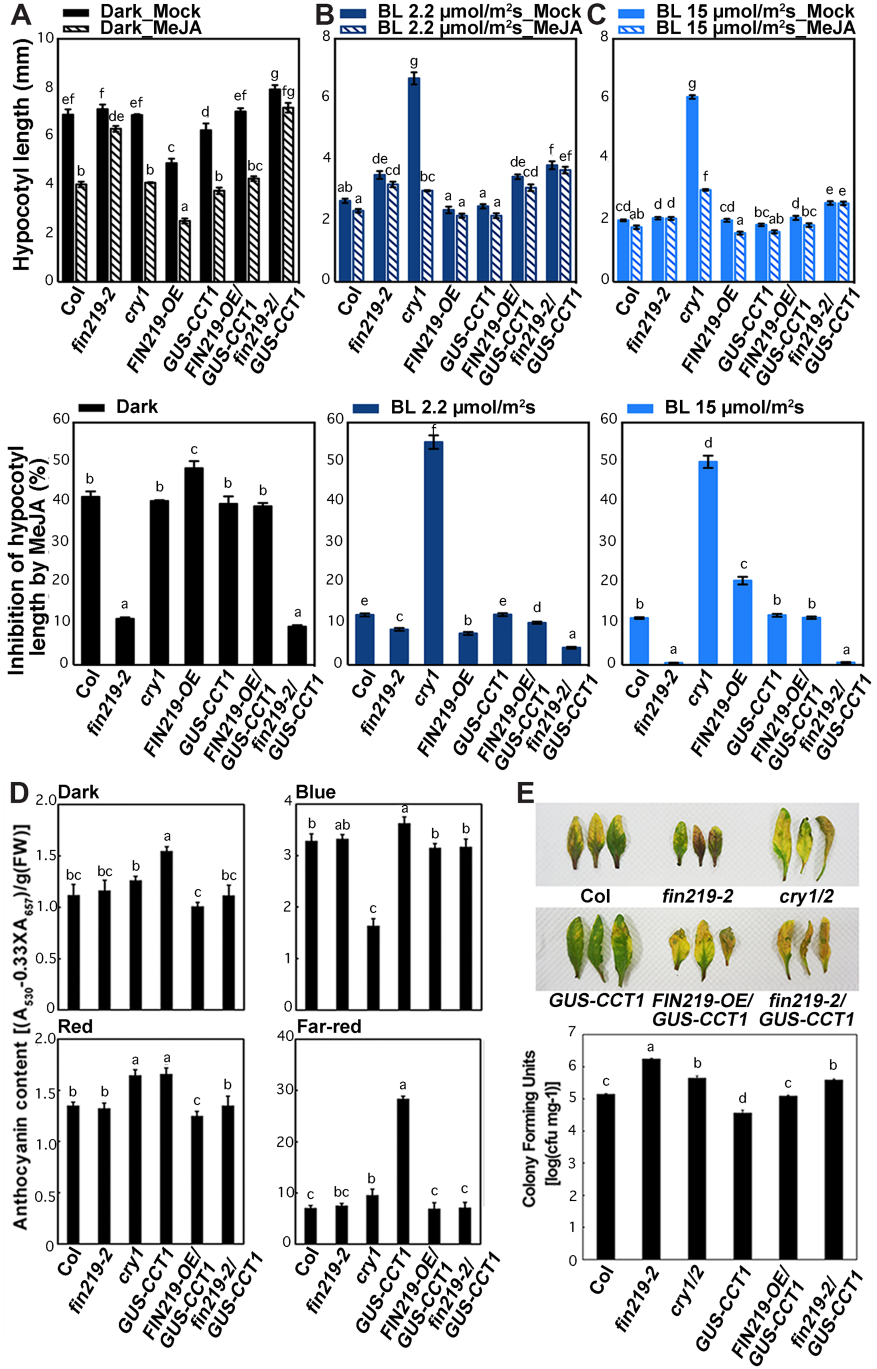
Changes in CRY1 levels affected MeJA-mediated physiological responses to various light conditions. The *cry1* mutant showed a hypersensitive response to MeJA-inhibited hypocotyl elongation under dark (A), low blue light (2 μmol•m^-2^•s^-1^) (B) and high blue light (15 μmol•m^-2^•s^-1^) as compared to the wild-type Col (C). Seedlings of wild-type Col, *fin219-2*, *cry1*, *FIN219-OE*, *GUS-CCT1*, *FIN219-OE/GUS-CCT1*, and *fin219-2/GUS-CCT1* were grown on GM plates without or with 50 μM MeJA for 3 days, then hypocotyl elongation with MeJA was analyzed. Different lowercase letters represent significant differences by one-way ANOVA at P <0.05. (D) Ectopic expression of *FIN219* reduced anthocyanin content in *GUS-CCT1* seedlings under different light conditions, including the dark. Seedlings were grown in various light conditions for 3 days, then anthocyanin content was determined. Different lowercase letters represent significant differences by one-way ANOVA at P <0.05. (E) Ectopic expression of *FIN219* reduced *GUS-CCT1* seedling resistance to *Pseudomonas syringae* pv. *tomato* (*Pst*.) DC3000 infection. Leaves of 5-week-old *Arabidopsis* plants grown under short-day conditions were inoculated with *Pst*. DC3000. Phenotypic response was shown in top panel and quantification of the bacterial number in each sample was in lower panel. Different lowercase letters represent significant differences by one-way ANOVA at P <0.05.

To associate the regulatory relation of FIN219 and CRY1 with physiological responses, we examined the responses of *CRY1*-related transgenic seedlings with or without MeJA treatment. The *cry1* mutant was more sensitive to MeJA-inhibited hypocotyl elongation than Col seedlings under blue light (Fig 6B and 6C). However, *GUS-CCT1* seedlings showed an opposite hypocotyl response to MeJA under blue light as compared to the dark (Fig 6A to 6C), which suggests that blue-light irradiation reduces the sensitivity of *GUS-CCT1* seedlings to MeJA. *FIN219* overexpression in *GUS-CCT1* seedlings (*FIN219-OE/GUS-CCT1*) could compromise *GUS-CCT1* phenotypic responses such as hypocotyl elongation and anthocyanin accumulation in the dark (Fig 6A and 6D). In contrast, FIN219 level in *GUS-CCT1* seedlings (*FIN219-OE/GUS-CCT1* or *fin219-2/GUS-CCT1*) affected the sensitivity of *GUS-CCT1* seedlings to MeJA-inhibited hypocotyl elongation under low and high blue light (Fig 6B and 6C). Moreover, *fin219-2/GUS-CCT1* seedlings showing a response to MeJA largely similar to *fin219-2* under dark, low and high blue light may reflect CRY1 response to MeJA with a requirement of FIN219, especially under the dark and high blue light (Fig 6A–6C). Furthermore, *GUS-CCT1* seedlings showed more anthocyanin accumulation under all light conditions, especially blue and FR light (Fig 6D); however, both *FIN219-OE/GUS-CCT1* and *fin219-2/GUS-CCT1* seedlings showed less anthocyanin accumulation than *GUS-CCT1* seedlings (Fig 6D). Therefore, regulation of anthocyanin accumulation under light conditions may involve more signaling regulators in addition to FIN219 and CRY1.

Arabidopsis cryptochromes have been implicated to participate in regulating stress responses [28–30]. We further examined the effect of FIN219 levels on the responses of *GUS-CCT1* leaves to the bacterium *Pseudomonas syringae* pv. *tomato* (*Pst*) DC3000. *GUS-CCT1* showed a resistant response to *Pst*. DC3000 infection as compared with the *cry1/2* double mutant and *fin219-2*, which showed a sensitive response as compared to Col-0 (Fig 6E). However, *FIN219-OE/GUS-CCT1* compromised the *GUS-CCT1* response, thereby resulting in a phenotype similar to wild-type Col-0. The *fin219-2/GUS-CCT1* was susceptible to *Pst*. DC3000, similar to the *cry1/2* double mutant. Thus, *GUS-CCT1* resistance to *Pst*. DC3000 may depend on optimal levels of FIN219.

## Discussion

**Fig 7:**
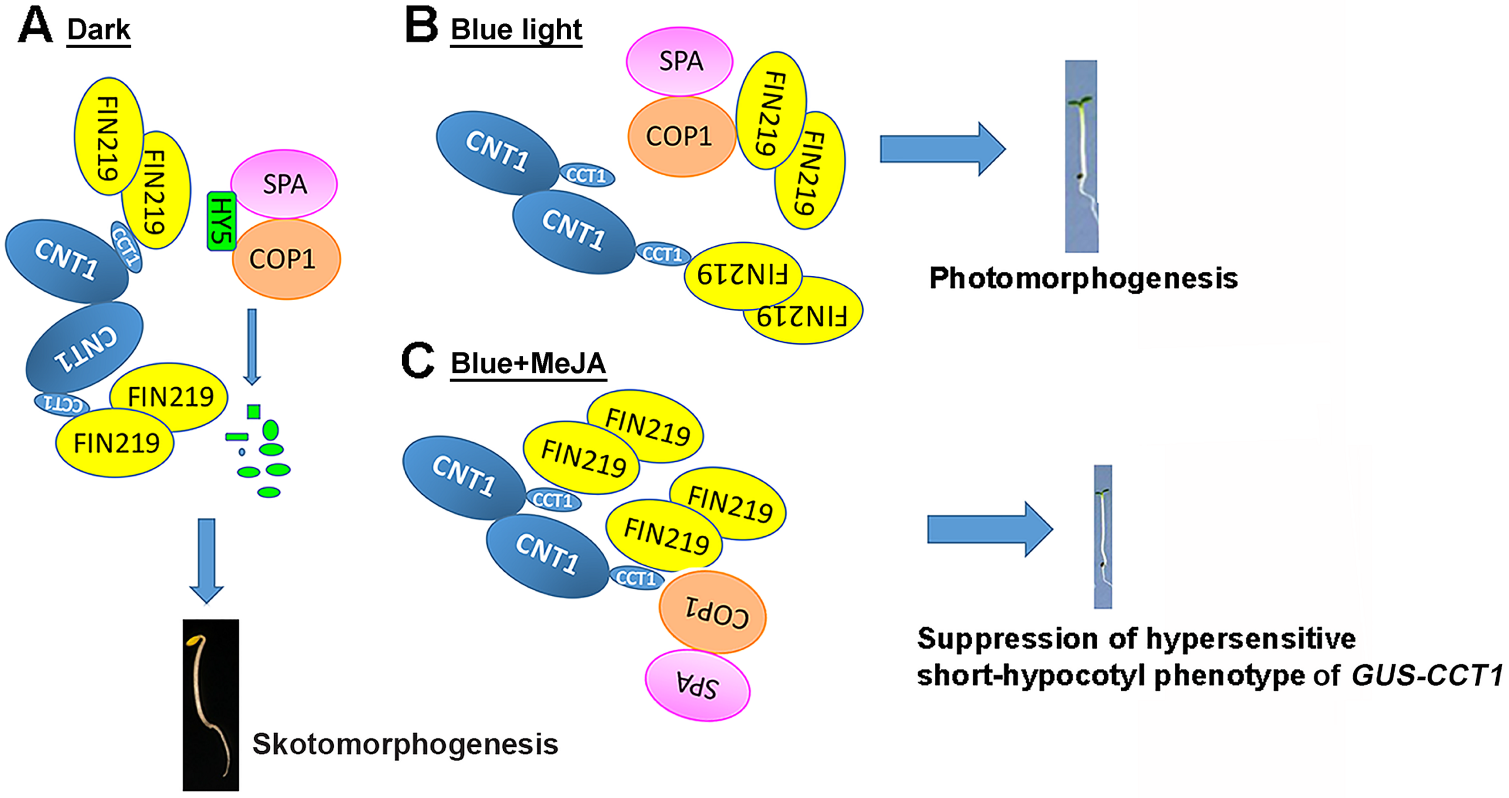
A model illustrates the effect of FIN219 levels on cry1 functions. (A) In the dark, FIN219 interacts with the C terminus of CRY1 (CCT1) to likely abolish CRY1 functions. Meanwhile, the COP1–SPA1 complex interacts with HY5, thereby degrading HY5 protein for skotomorphogenic development of Arabidopsis seedlings. (B) In blue light, FIN219 interacts closely with CRY1 as well as COP1, resulting in cry1-mediated photomorphogenesis. (C) In the presence of blue light, induction of FIN219 by MeJA strongly interacts with CCT1, and weakly with COP1–SPA1 complex, leading to a rescued phenotype of *GUS-CCT1* seedlings by suppressing hypersensitive short-hypocotyl phenotype.

Our studies revealed the cross talk between blue light and JA signaling in regulating photomorphogenic responses in Arabidopsis such as hypocotyl elongation and anthocyanin accumulation as well as bacterial pathogen response. In the dark, FIN219/JAR1 interacted with the C terminus of CRY1 (CCT1) and the full-length CRY1 (Fig 5D and 5E). COP1 and suppressor of phytochrome A-105 1 (SPA1) interacts with HY5 in the dark [25, 32], which leads to the degradation of HY5 and a skotomorphogenic development of Arabidopsis seedlings (Fig 7A). In contrast, FIN219/JAR1 strongly interacts with COP1 and CRY1 under blue light, thereby leading to CRY1-mediated photomorphogenesis (Fig 7B). Under blue light and MeJA treatment, FIN219/JAR1 greatly interacts with CCT1 and weakly with COP1, likely enhancing COP1 access to CCT1, which results in the suppression of hypersensitive short-hypocotyl phenotype of *GUS-CCT1* (Fig 7C), leading to the outcomes with a rescued phenotype of *FIN219-OE/GUS-CCT1* under blue light (Fig 1C and 1D).

The cross talks between light and JA signaling, especially FR light and JA signaling, are being revealed [12, 33, 34]. Phytochrome chromophore-mediated signaling and the JA signaling pathway mutually regulate each other in an antagonistic manner [35, 36]. Moreover, phytochrome inactivation by FR light greatly reduces plant sensitivity to jasmonates [35]. phyA was shown to be required for JA- and wound-mediated JAZ1 degradation [12]. As well, phyA in rice requires JA for photo-destruction [37]. Therefore, phyA- and JA-mediated signaling regulate each other to modulate seedling development. In addition, AtMYC2/JIN1, a basic helix-loop-helix transcription factor, can bind to the Z- and G-box light-responsive elements of light-regulated promoters and acts as a vital regulator in light, abscisic acid (ABA) and JA signaling pathways in Arabidopsis [38]. LeMYC2 in tomato functions in a similar manner to regulate photomorphogenesis and tomato growth in response to the combined effects of blue light, ABA and JA signaling [39]. Recent studies also indicated that rice *phyAphyC* mutant seedlings greatly increased JA and JA-Ile levels as compared with the wild type in response to blue light [40], which suggests that phyA and phyC in rice may redundantly and negatively modulate JA biosynthesis under blue light. Indeed, we found that the blue-light photoreceptor CRY1 negatively regulated the levels of FIN219, a JA-conjugating enzyme for the formation of JA-Ile [27], under blue light (Fig 2A).

How FIN219/JAR1 regulates cry1-mediated blue light signaling is intriguing. Ectopic expression of *FIN219* in wild-type Col produced a hypersensitive short-hypocotyl phenotype under blue light (Fig 1, S1 Fig), which suggests functional roles of FIN219 in blue light-inhibited seedling development. This speculation is substantiated by results showing that ectopic expression of *FIN219* in different *cry* mutants produced a long-hypocotyl phenotype as well as a FIN219-rescued GUS-CCT1 phenotype under blue light (Fig 1). Further evidence revealed greatly enhanced FIN219 and GUS-CCT1 interaction with MeJA treatment under blue light. Moreover, FIN219 in *GUS-CCT1* seedlings strongly interacted with COP1 under blue light (Fig 5D), which may restrict COP1 activity and release HY5, thereby leading to photomorphogenesis. In addition, CCT1 interacting with SPA1 depends on blue light, which leads to reduced COP1 and SPA1 interaction as well as COP1 E3 ligase activity [26, 41]. FIN219 and COP1 interaction as well as CCT1 and SPA1 interaction under blue light are likely highly involved in photomorphogenic development of seedlings. As FIN219 level increases, it becomes associated with CRY1, which suppresses CRY1 functions likely by competing out SPA1 binding with CRY1, thereby producing longer hypocotyls under blue light as compared with GUS-CCT1 seedlings (Fig 1).

In addition, FIN219 itself is mainly localized in the cytoplasm [3]. However, it could be localized in the nucleus when associated with nuclear-localized proteins. CRY1 is more specifically localized in the nucleus in response to blue light (Fig 5C, bottom panel) [31, 42]. Here, we found that FIN219 interacted with CRY1 in the nucleus under blue light (Fig 5C, bottom panel). However, FIN219 interacted with COP1 under blue light likely in the cytoplasm (Fig 5C, bottom panel). Our previous studies showed that FIN219 overexpression resulted in COP1 accumulation in the cytoplasm even in the dark [18]. In contrast, ethylene under light conditions can trigger COP1 accumulation in the nucleus [6, 43]. Thus, JA and ethylene in addition to the light effect may antagonize each other to modulate the subcellular location of COP1 in regulating plant photomorphogenic development. Therefore, FIN219 levels need to be tightly regulated to modulate seedling development in response to various light conditions. This conclusion is also consistent with *GUS-CCT1*-mediated responses such as anthocyanin accumulation (Fig 6D) and resistance to *Pst*. DC3000 (Fig 6E).

In addition, FIN219 levels were greater in *cry1* mutant than Col-0 seedlings and substantially greater in *FIN219-OE*/*cry1cry2* than *cry1cry2* mutant or *FIN219-OE* seedlings (Fig 2A); however, the hypocotyl phenotype of *FIN219-OE*/*cry1cry2* and the *cry1cry2* mutant was similar (Fig 1A and 1B), which suggests that FIN219 function in blue light may require functional CRY1 and CRY2. Moreover, the increased accumulation of FIN219 in *FIN219-OE*/*cry1cry2* seedlings might be in an inactive form of FIN219 involving posttranslational modifications such as phosphorylation, which remains to be further elucidated.

Our study revealed an antagonistic regulation between CRY1 and FIN219/JAR1 in modulating blue light-inhibited hypocotyl elongation in Arabidopsis and their protein levels under blue light (Figs 1 and 2). FIN219/JAR1 function requires functional CRY1 in regulating hypocotyl elongation. Moreover, CRY1 functions require an optimal level of FIN219/JAR1 to optimize photomorphogenic development and stress responses such as anthocyanin accumulation and pathogen resistance (Fig 6). Thus, the cross talks between blue light and JA signaling pathways are critical in regulating seedling development and biotic stress responses in Arabidopsis.

## Materials and methods

### Plant materials and growth conditions

Throughout this study, the wild-type plant is *Arabidopsis thaliana* ecotype Columbia. Blue-light photoreceptor mutants *cry1* (*cry1-304*) [19], *cry2* (*cry2-1*) [44] and the *cry1cry2* (*cry1-304cry2-1*) [45] double mutant were in the Columbia ecotype. The *fin219* knockout mutant (*fin219-2*) in the Columbia ecotype was the T-DNA insertion line SALK_059774 obtained from ABRC [18]. Transgenic *GUS-CCT1* was established previously [21]. The overexpression of *FIN219* (*FIN219-OE*) in a Columbia background was established previously [3]. *FIN219-OE*/*cry1*, *cry2* or c*ry1cry2* and *FIN219-OE*/*GUS-CCT1* were generated by crossing *FIN219-OE* with respective mutants. The resulting progenies were selected with 100 μg/ml gentamycin (MdBio, Taipei) for *FIN219-OE* and with genotyping for *cry1* and *cry2* mutants by using primer sequence pairs: *cry1-304*, 5'- CATGAGCTCATGTCTGGTTCTGTATCTGGTT -3' and 5'- CATGTCGACTGAAAGCGCTTCATGAA -3'; *cry2-1*, 5'- CATGAGCTCATGAAGATGGACAAAAAGAC -3' and 5'- CATGGTACCAGCTTTAGCTAGTAGCTCACG -3'. Hygromycin 25 μg/ml (MdBio, Taipei) was also used for selecting *FIN219-OE/GUS-CCT1*. We crossed a *GUS-CCT1* transformant with *fin219-2* and an inducible *FIN219*-overexpressing line *PGR219* (*pGR*:*FIN219*). F3 and F4 seedlings were selected with 25 μg/ml hygromycin and 50 μg/ml kanamycin for *PGR219/GUS-CCT1*, and 50 μg/ml kanamycin and genotyping by using primer sequence pairs for FIN219-LP-F: 5’-CTACATTTTTGCTGCTCCGTC-3’; FIN219-RP-R: 5’-AAAAGCAGTGCGAAACAGTTG-3’; and LBb1.3: ATTTTGCCGATTTCGGAAC for *fin219-2/GUS-CCT1*. Seeds were surface-sterilized with 20% bleach containing 0.5% Tween-20 [18] and sown on GM agar plates containing 0.3% sucrose for phenotype analysis and MeJA (50 μM) for its effects and molecular analysis.

### Protein extraction and Western blot analysis

Seedlings grown in continuous blue light (2 μmol m^-2^ s^-1^) or far-red light (3 μmol m^-2^ s^-1^) for 3 days were harvested for protein extraction. For MG132 treatment, *FIN219OE/GUS-CCT1* seedlings were grown in the dark for 2 days, then transferred to blue light with or without MG132 (50 μM) for 15 or 60 min or 24 h. Total proteins were extracted with extraction buffer (50 mM Tris-HCl, pH7.5, 150 mM NaCl, 10 mM MgCl_2_, 0.1% NP-40, 1 mM PMSF and 1X protease inhibitor) as described [18]. Total proteins, 50 μg, were loaded in each lane and separated on 10% SDS-PAGE and transferred to a PVDF membrane (Millipore). Protein gel blot analysis involved standard methods and bands were detected with GUS, COP1, HY5 or FIN219 (monoclonal) antibodies [7].

### RT-PCR analysis

Total RNA was isolated from 4-day-old seedlings grown under the dark or blue light. For high-fidelity RT-PCR, 5 μg total RNA was treated with RQ1 DNase I (Promega, Madison, WI) according to the manufacturer’s instructions to remove possible DNA contamination. Then 3 μg of DNase-treated total RNA underwent reverse transcription at 42°C for 1 h with Ready-To-Go RT-PCR beads (Amersham-Pharmacia Biotech, Rome, Italy) and was inactivated at 95°C for 10 min. GUS-CCT1 was amplified by using GUS3’-F and CRY1-R primers from 1 μl of 50 μl of cDNA by PCR for 30 cycles (95°C, 30 s; 55°C, 30 s; 72°C, 40 s) with a Peltier thermal cycler (MJ Research, Watertown, MA); c-myc-FIN219 for 35 cycles (95°C, 30 s; 52°C, 30 s; 72°C, 150 s) with Myc5’-F and FIN219-R-*Xho*I primers, and ubiquitin10 for 28 cycles (95°C, 30 s; 60°C, 30 s; 72°C, 40 s) with UBQ10-F and UBQ10-R primers.

### Plasmid construction and pull-down assays

*pGEX-4T-1*, *FIN219FL/pGEX-4T-1*, *FIN219N300/pGEX-4T-1* and *FIN219C274/pGEX-4T-1* were created as described [18]. Fragments of CRY1, CNT1, and CCT1 PCR-amplified with gene-specific primers were cloned into *Sal*I and *Sac*I sites of *pCal-n* (Stratagene, La Jolla, CA). Resulting constructs (*CRY1/pCal-n*, *CNT1/pCal-n*, and *CCT1/pCal-n*) were used for expressing the recombinant fusion proteins CBP-CRY1, CBP-CNT1, and CBP-CCT1, respectively. The recombinant plasmids were transformed into *E*. *coli* BL21, then induced with 0.1 mM isopropylthio-β-galactoside (IPTG) at 25°C overnight (*pGEX-4T-1*, *FIN219FL/pGEX-4T-1* and *CCT1/pCal-n*) or 1 mM IPTG at 37°C for 4 h (*FIN219N300/pGEX-4T-1*, *CRY1/pCal-n*, and *CNT1/pCal-n*) for expression in *E*. *coli* hosts BL21 (DE3) codon plus (*pGEX-4T-1*, *FIN219FL/pGEX-4T-1*, *FIN219N300/pGEX-4T-1*, and *CRY1/pCal-n*) or *E*. *coli* BL21 (DE3) pLysS (*FIN219C274/pGEX-4T-1*, *CNT1/pCal-n*, and *CCT1/pCal-n*). Recombinant fusion proteins were purified with use of GSH sepharose (Amersham-Pharmacia Biotech, Rome, Italy) for GST, GST-FIN219 full-length, and GST-FIN219-C274; calmodulin affinity resin (Stratagene, LaJolla, CA) for CBP-CCT1; or electroelusion (Bio-Rad, Hercules, CA) for GST-FIN219-N300, CBP-CRY1, and CBP-CNT1 according to the manufacturer’s procedures. All recombinant fusion proteins were precipitated with acetone and resuspended in PBS solution (80 mM Na_2_HPO_4_, 20 mM NaH_2_PO_4_, 100 mM NaCl). Then recombinant fusion proteins were concentrated by using Amicon Ultra-4 (Millipore, Billerica, MA) for downstream analysis.

An amount of 5 μg purified recombinant protein was mixed in 500 μl interaction buffer (50 mM Tris-HCl, pH 7.5, 10 mM MgCl_2_, 100 mM NaCl, 1 mM phenylmethylsulfonyl fluoride, 0.04% NP-40) with 1 x protease inhibitors (Invitrogen, Carlsbad, CA), then incubated at 4°C at 30–40 rpm for 1 h. Well-equilibrated GSH sepharose was added to the mixture and incubated at 4°C for another hour. After centrifugation (500 g for 5 min), the pellet was washed with interaction buffer and analyzed by protein gel blot analysis.

### Co-immunoprecipitation analysis

Seedlings grown in the dark or continuous blue light for 3 days were ground with extraction buffer as described [18]. Co-immunoprecipitation analysis followed the manual (GE, USA). A total of 2 mg protein was mixed with beads and incubated at 4°C for 4 h, then washed three times with TBST washing buffer (TBS with 0.05% Tween-20, pH7.5). Pellets were analyzed by SDS-PAGE and protein gel blot analysis.

### Protoplast transfection and bimolecular fluorescence complementation (BiFC) analysis

Arabidopsis mesophyll protoplast isolation and transfection were as described previously [18]. We constructed BiFC plasmids as described [18]. The full-lengths of CRY1 or COP1, and FIN219 were cloned into 35p-YFP-N155/pRTL2 and 35p-YFP-C84/pRTL2, respectively. The nuclei of protoplasts were marked with NLS-mCherry cloned into the pEarlyGate 100. All fluorescence images were obtained by use of a Nikon CI-L/Nikon Ri2 Cooling fluorescence microscope and processed by use of Adobe Photoshop.

### Anthocyanin extraction and quantification

For anthocyanin determination in seedlings, harvested samples were weighed and ground in liquid nitrogen, and total plant pigments were extracted overnight in 300 μl 1% HCl in methanol. After the addition of 200 μl H_2_O, chlorophyll was separated from anthocyanin by extraction with an equal volume of chloroform. The content of anthocyanin in the upper phase was quantified by spectrophotometry (A_530_-A_657_) and normalized to the fresh weight of seedlings [11].

### Pathogen infection assays

Bacteria (Pseudomonas syringae pv. tomato DC3000) grown on King’s medium B [38] containing 50 μg/ml rifampicin for 2 days at 28°C were diluted with appropriate 10 mM of MgCl_2_ solution (1X10^6^ cfu ml^-1^, OD_600_ = 0.002). For infiltration inoculation, bacterial suspension cells were injected into leaves of 5-week-old Arabidopsis plants grown under short-day conditions through stomatal pores on the leaf surface by using a needle-less syringe. After 2 days, infected leaves were collected, weighed and grounded with plastic pestles. To assay bacterial populations, samples were serially diluted with 10 mM MgCl_2_ and plated on KB solid medium at 28°C for 2 days to count colony units.

### Statistical analysis

One-way ANOVA was used to quantify hypocotyl length, root length, chlorophyll content, anthocyanin accumulation and bacteria number by using SAS 9.3.

## Supporting information

S1 Fig Ectopic expression of *FIN219* in wild-type Col-0 resulted in a hypersensitive short-hypocotyl phenotype under blue light. (A) Transgenic seedlings harboring a *FIN219* overexpression construct showed a hypersensitive short-hypocotyl phenotype under blue light. Seedlings of wild-type Col-0, *fin219-2*, and *FIN219* overexpression line (*FIN219-OE*) were grown in continuous blue light (2.2 μmol•m^-2^•s^-1^) for 3 days, then underwent phenotype examination and imaging. (B) Quantification of hypocotyl lengths of seedlings shown in (A) (n = 30). Different lowercase letters represent significant differences by one-way ANOVA at P <0.05.
(TIF)

S2 Fig CRY1 negatively regulated FIN219 levels under continuous far-red light. Western blot analysis of FIN219 protein level in wild-type Col-0, *cry1*, *cry2* mutants and different transgenic seedlings grown under far-red light for 3 days. The signal was detected by FIN219 polyclonal antibody. Far-red light: 3 μmol•m^-2^•s^-1^. The asterisk (*) indicates nonspecific bands. RPN8 was a loading control.
(TIF)

S3 Fig *GUS-CCT1* transcripts were detected in transgenic seedlings of *FIN219-OE/GUS-CCT1* under blue light. RT-PCR analysis of the transgenic seedlings as shown in Fig 2B. Total RNAs were extracted from transgenic seedlings grown under blue light for 3 days and subjected for RT-PCR analysis. *Ubiquitin 10* (*UBQ10*) was used as an internal control.
(TIF)

S4 Fig FIN219-down-regulated COP1 levels under blue light were modulated by GUS-CCT1. Western blot analysis of COP1 protein levels in wild-type Col-0, *fin219-2*, *cry1*, *cop1-4* mutants and various transgenic seedlings grown under blue light for 3 days. The signal was detected by COP1 polyclonal antibody. Each lane contains 100 μg total proteins. The *cop1-4* mutant without the full-length of COP1 was used as a negative control. Blue light: 2 μmol•m^-2^•s^-1^. The asterisk (*) indicates nonspecific bands. The number below the blot indicates the relative expression level. COP1 level in Col-0 is set to 1. The image of a coomassie blue-stained gel below the blot was used as loading control.
(TIF)
