# Correction: FIN219/JAR1 and cryptochrome1 antagonize each other to modulate photomorphogenesis under blue light in *Arabidopsis*

**DOI:** 10.1371/journal.pgen.1007606

**Published:** 2018-08-16

**Authors:** Huai-Ju Chen, Tsu-Yu Fu, Shao-Li Yang, Hsu-Liang Hsieh

There is an error in panel A of Fig 2. Specifically, the sample order should read ‘Col-0, *FIN219-OE*, *cry1*, *FIN219-OE/cry1*, *cry2*, *FIN219-OE/cry2*, *cry1cry2*, *FIN219-OE/cry1cry2*’, not ‘Col-0, *FIN219-OE*, *cry1*, *cry2*, *FIN219-OE/cry1*, *cry1cry2*, *FIN219-OE/cry2*, *FIN219-OE/cry1cry2*’. The authors have provided a corrected version here.

**Fig 2:**
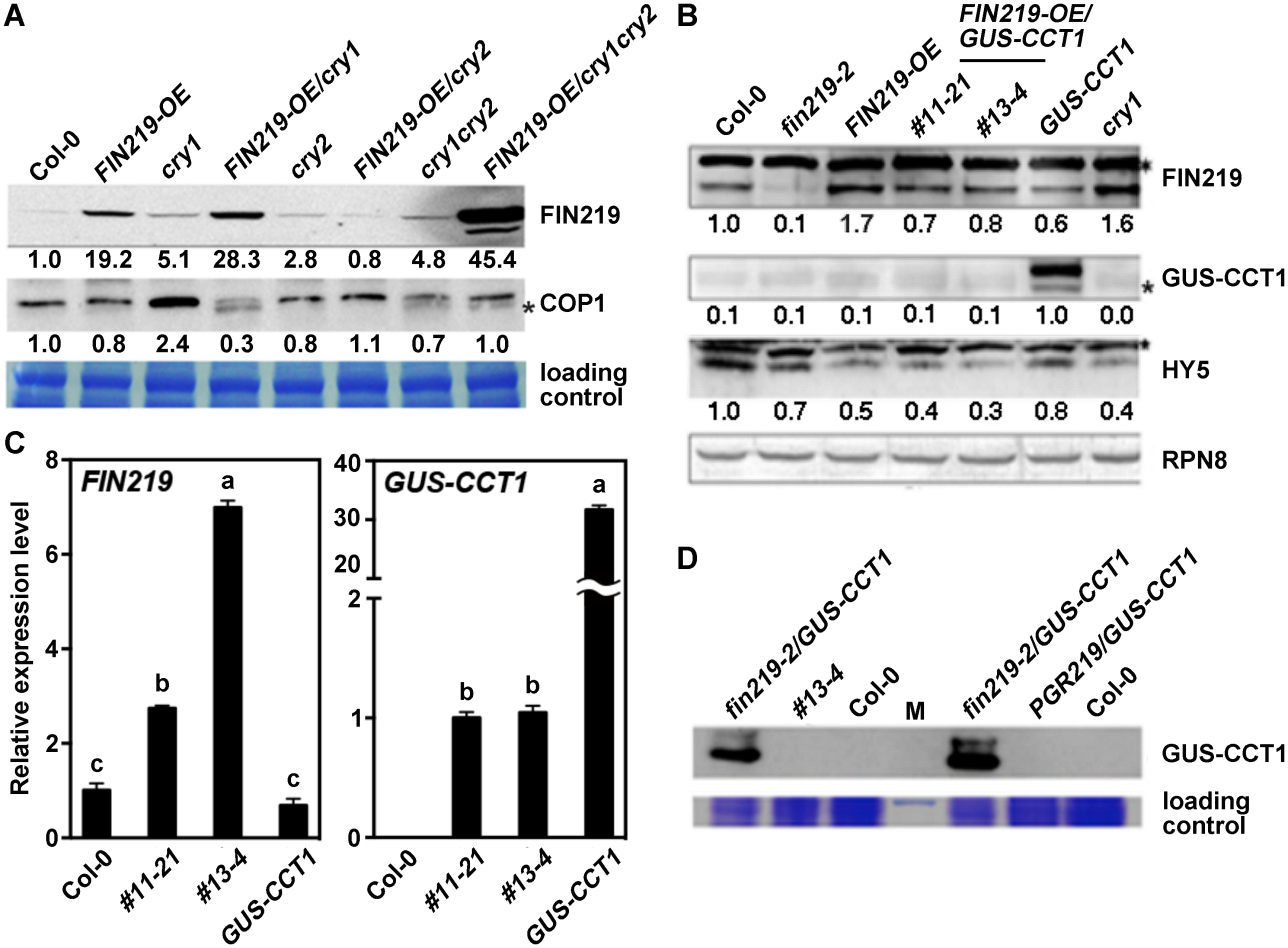
FIN219 and CRY1 antagonize each other under blue light. (A) CRY1 and CRY2 negatively regulate FIN219 protein level under blue light. Western blot analysis of FIN219 protein level in wild-type Col-0, *cry1*, *cry2* mutants and transgenic seedlings grown under blue light. The signal was detected by FIN219 monoclonal antibody. Blue light: 2.2 μmol•m^-2^•s^-1^. The number below each blot represents the level of the indicated protein. The level of wild-type Col-0 was arbitrarily set to 1. The asterisk (*) indicates nonspecific bands. (B) *FIN219* overexpression in *GUS-CCT1* seedlings abolishes GUS-CCT1 fusion protein in blue light. Western blot analysis of protein levels in Col-0, *fin219-2*, *FIN219* overexpression line (*FIN219-OE*), *FIN219-OE/GUS-CCT1*, *GUS-CCT1* and *cry1* seedlings grown in blue light for 3 days. The blots were detected by antibodies against FIN219 and GUS-CCT1 and HY5. Blue light: 2.2 μmol•m^-2^•s^-1^. RPN8 was a loading control. The asterisk (*) indicates nonspecific bands. The number below each blot represents the level of the indicated protein. The level of wild-type Col-0 was arbitrarily set to 1. (C) *GUS-CCT1* transcripts detected in transgenic seedlings of *FIN219-OE/GUS-CCT1* under blue light. Quantitative Real-time PCR (qPCR) analysis of transgenic seedlings shown in B. Total RNAs were extracted from transgenic seedlings shown in the figure and subjected for qPCR analysis. *Ubiquitin 10* (*UBQ10*) was an internal control. (D) GUS-CCT1 fusion proteins were stable in *fin219-2/GUS-CCT1* seedlings under blue light. Western blot analysis of GUS-CCT1 level in Col-0, *FIN219-OE/GUS-CCT1* (#13–4), *fin219-2/GUS-CCT1* and *PGR219/GUS-CCT1* seedlings grown in blue light for 4 days. Total proteins extracted from seedlings were probed with GUS antibody. M, protein size markers.
