# Supplementary figures and images for: FIN219/JAR1 and cryptochrome1 antagonize each other to modulate photomorphogenesis under blue light in Arabidopsis
Source: PLoS Genet. 2018 Mar 21;14(3):e1007248. doi: 10.1371/journal.pgen.1007248 (PMC5880400; doi:10.1371/journal.pgen.1007248)

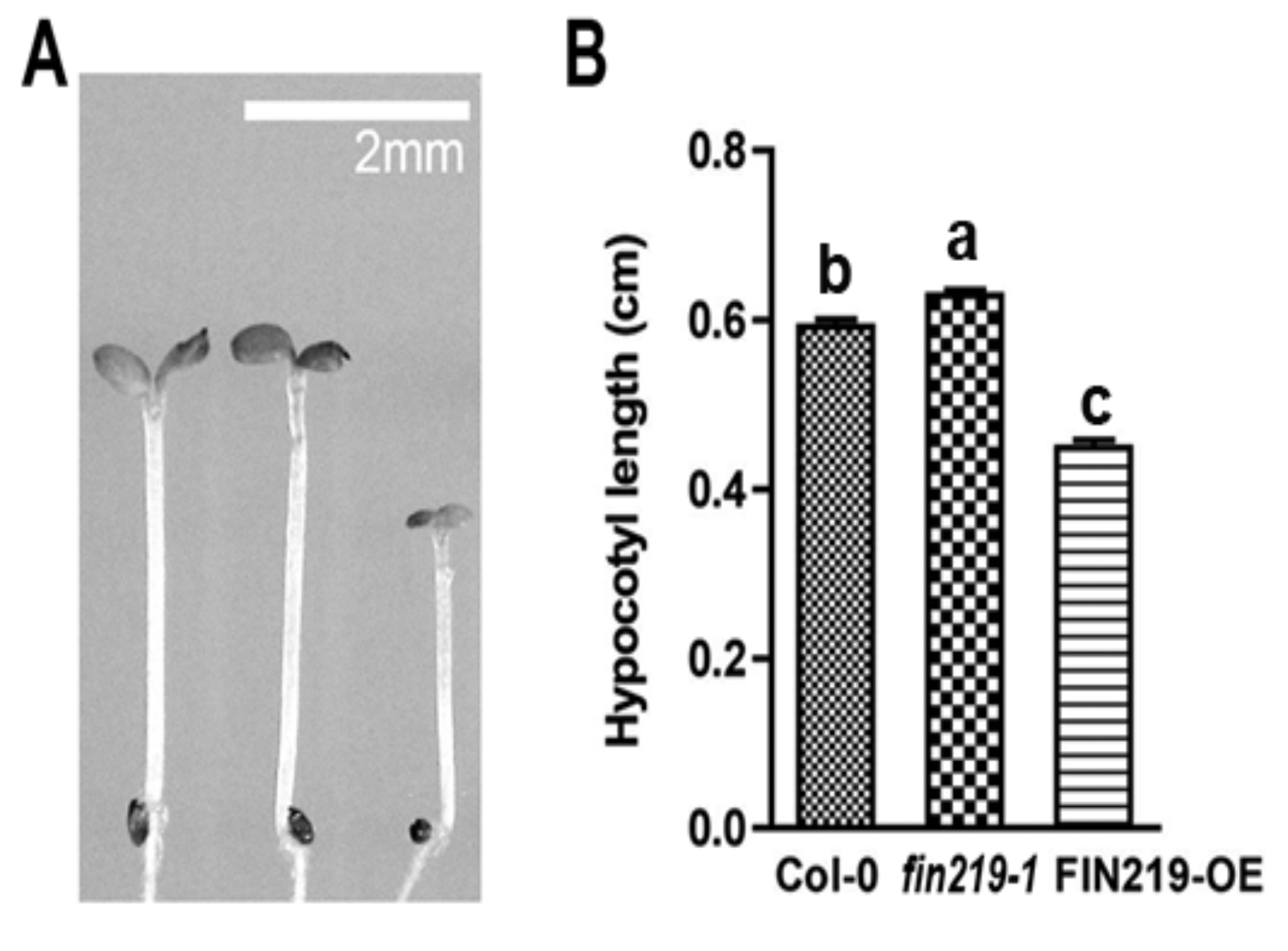

Supplement: S1 Fig — (A) Transgenic seedlings harboring a FIN219 overexpression construct showed a hypersensitive short-hypocotyl phenotype under blue light. Seedlings of wild-type Col-0, fin219-2, and FIN219 overexpression line (FIN219-OE) were grown in continuous blue light (2.2 μmol•m-2•s-1) for 3 days, then underwent phenotype examination and imaging. (B) Quantification of hypocotyl lengths of seedlings shown in (A) (n = 30). Different lowercase letters represent significant differences by one-way ANOVA at P <0.05. (TIF) [file pgen.1007248.s001.tif]

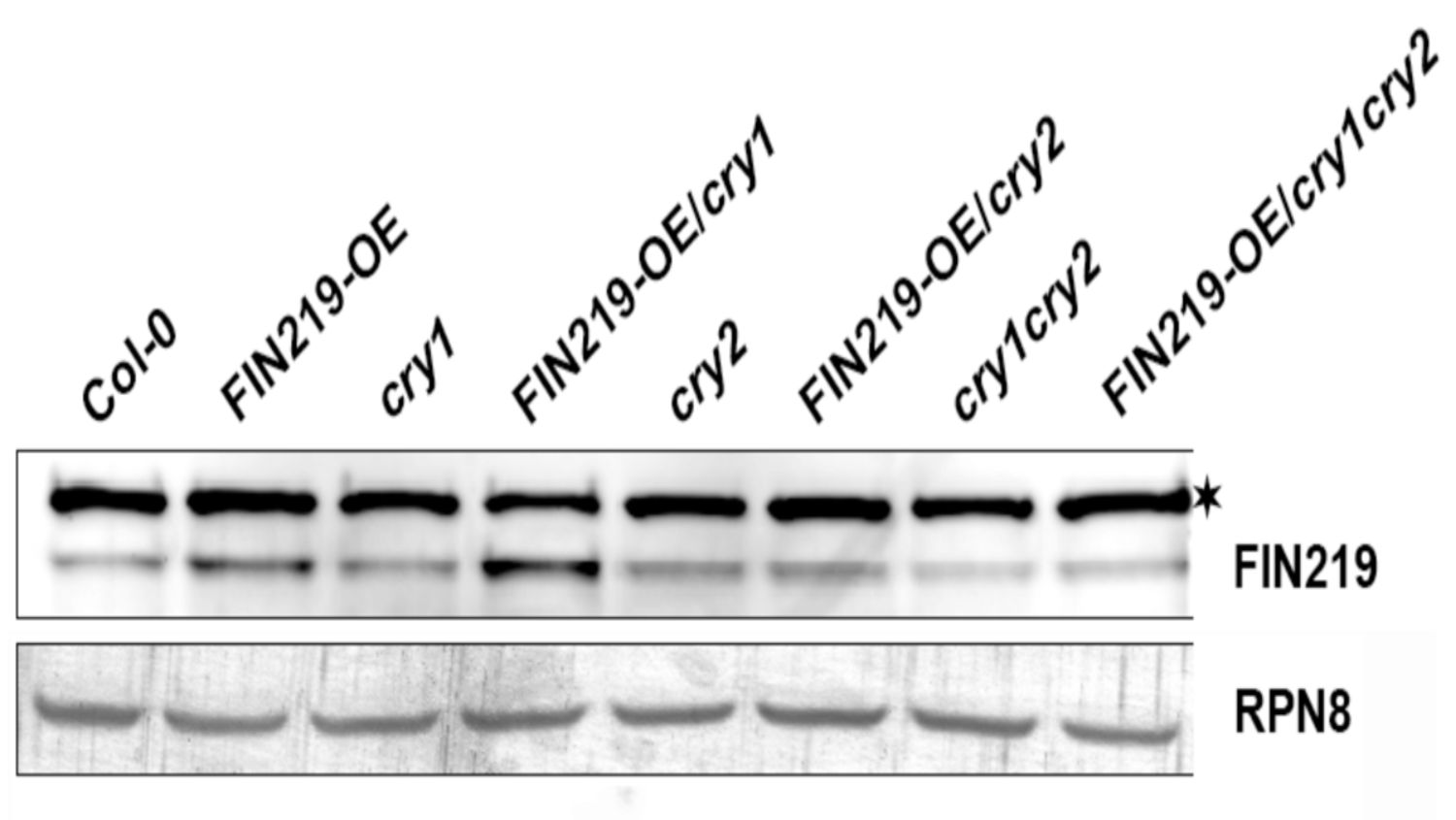

Supplement: S2 Fig — Western blot analysis of FIN219 protein level in wild-type Col-0, cry1, cry2 mutants and different transgenic seedlings grown under far-red light for 3 days. The signal was detected by FIN219 polyclonal antibody. Far-red light: 3 μmol•m-2•s-1. The asterisk (*) indicates nonspecific bands. RPN8 was a loading control. (TIF) [file pgen.1007248.s002.tif]

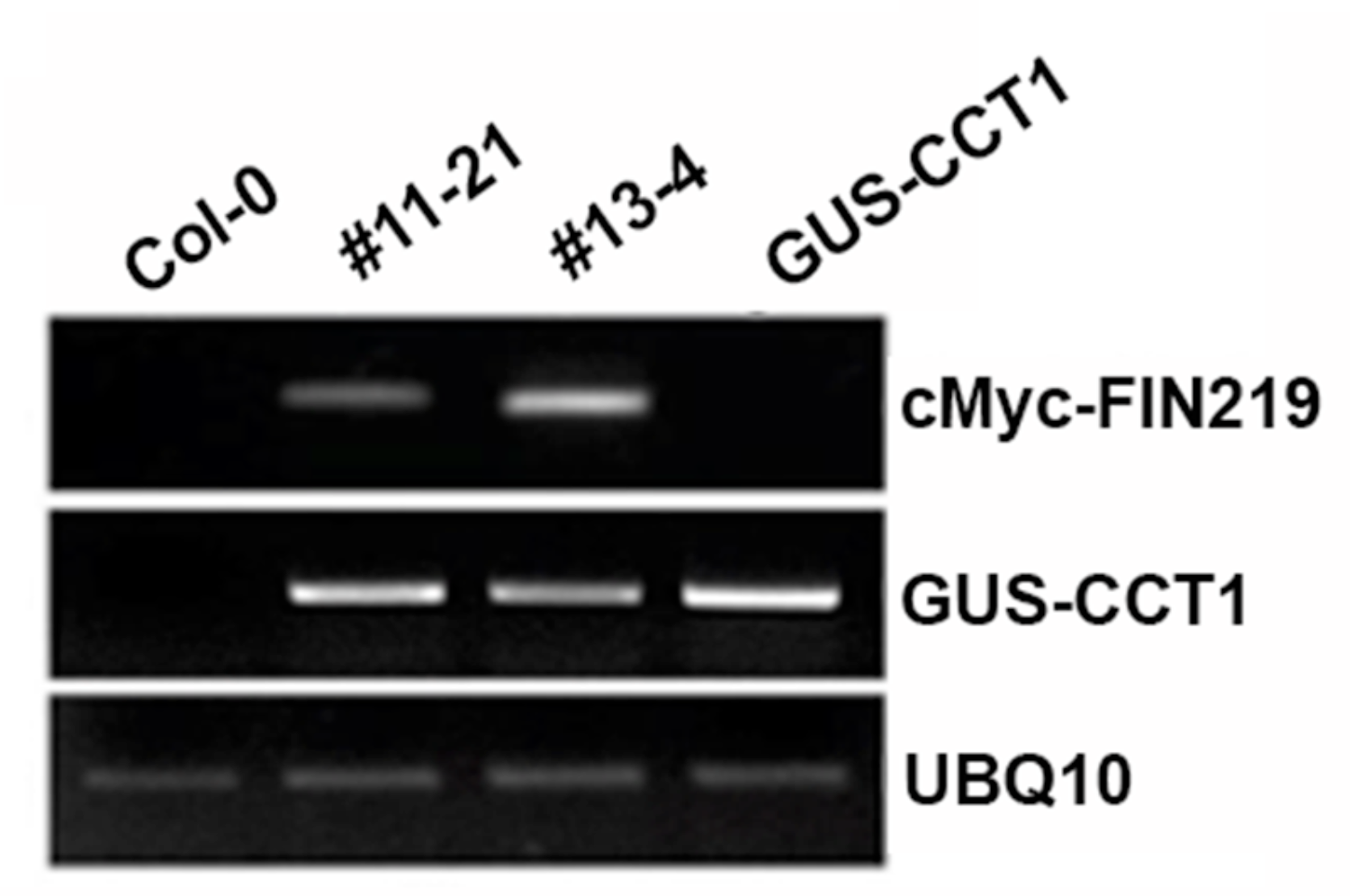

Supplement: S3 Fig — RT-PCR analysis of the transgenic seedlings as shown in Fig 2B. Total RNAs were extracted from transgenic seedlings grown under blue light for 3 days and subjected for RT-PCR analysis. Ubiquitin 10 (UBQ10) was used as an internal control. (TIF) [file pgen.1007248.s003.tif]

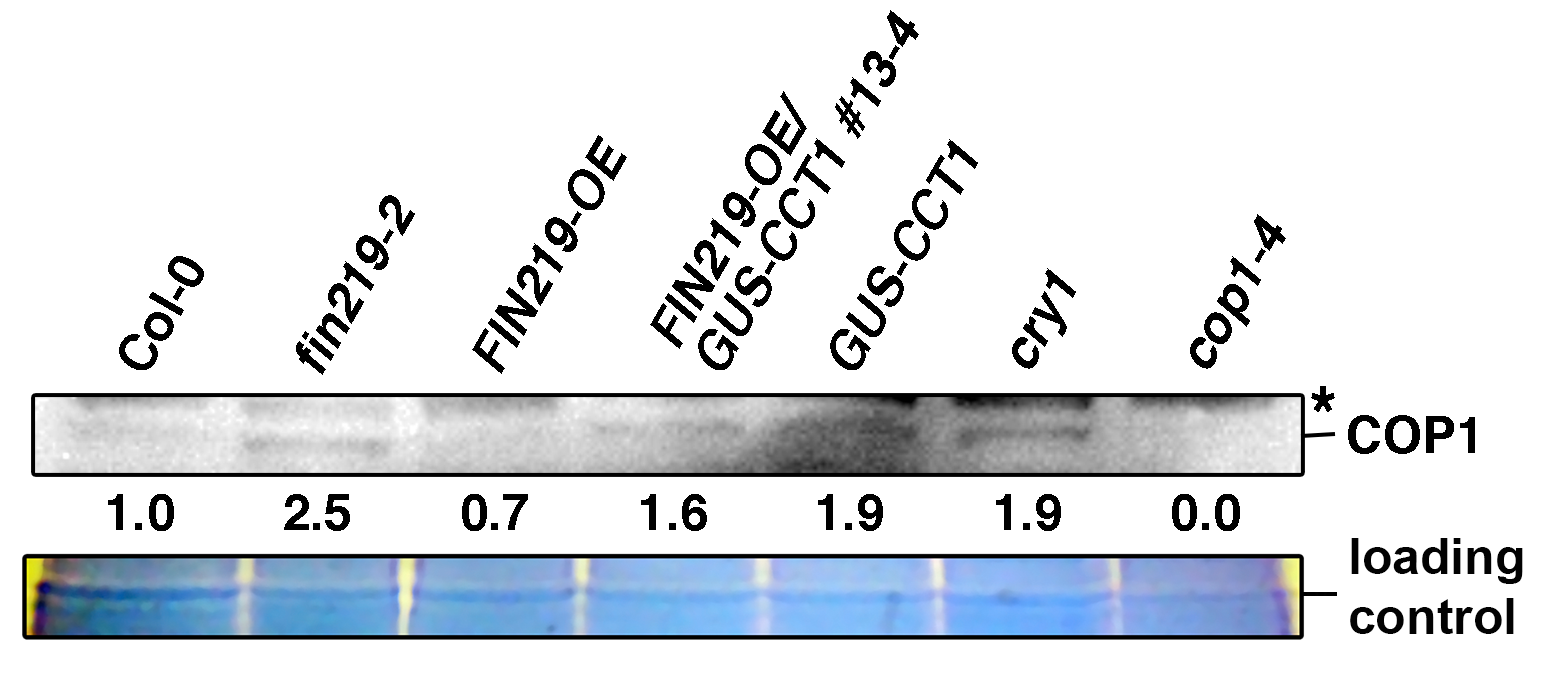

Supplement: S4 Fig — Western blot analysis of COP1 protein levels in wild-type Col-0, fin219-2, cry1, cop1-4 mutants and various transgenic seedlings grown under blue light for 3 days. The signal was detected by COP1 polyclonal antibody. Each lane contains 100 μg total proteins. The cop1-4 mutant without the full-length of COP1 was used as a negative control. Blue light: 2 μmol•m-2•s-1. The asterisk (*) indicates nonspecific bands. The number below the blot indicates the relative expression level. COP1 level in Col-0 is set to 1. The image of a coomassie blue-stained gel below the blot was used as loading control. (TIF) [file pgen.1007248.s004.tif]
